# Leadership potential of physicians in a public teaching hospital in the city of São Paulo

**DOI:** 10.31744/einstein_journal/2019GS4191

**Published:** 2019-01-17

**Authors:** Alexandre Fioranelli, Charles Schmidt, Nelson Wolosker, Valter Castelli, Dafne Braga Diamante Leiderman, Luiz Arnaldo Szutan

**Affiliations:** 1 Faculdade de Ciências Médicas , Santa Casa de São Paulo , São Paulo , SP , Brazil .; 2 Hospital Israelita Albert Einstein , São Paulo , SP , Brazil .

**Keywords:** Leadership, Work performance, Physicians, Hospital, teaching, Hospitals, voluntary, Liderança, Desempenho profissional, Médicos, Hospital de ensino, Hospital voluntário

## Abstract

**Objective:**

To analyze the leadership potential of physicians in a public hospital in the city of São Paulo.

**Methods:**

A descriptive pilot study, in which 40 assistant physicians and medical residents were randomly selected to receive an electronic invitation of the company *Caliper Estratégias Humanas do Brasil* . To those who accepted it, a link was sent to fill out a personality evaluation focused on the work, comprising 112 alternatives related to 21 domains of 4 performance areas. According to the Caliper Profile Questionnaire, the ipsative measures expressed as a percentage are distributed on a Likert scale, and three categories are established based on behavioral tendencies at work: need for improvement, moderate and high potential.

**Results:**

A total of 47.5% of physicians invited accepeted taking part in the study. Regarding to leadership, the need for improvement was over 30% among the evaluated physicians. In the interpersonal relationship analysis, only 18.4% of assistant physicians and 37% of medical residents required improvement. The percentage of physicians who needed improvement in problem-solving and decision-making was similar among the assistant and resident physicians (12.6% *versus* 14%). In the evaluation of personal organization and time management, we obtained similar percentages in assistant physicians and residents who needed improvement (14% in both groups). High potential leadership was observed in these domains (18.4% and 20% for assistant physicians and residents, respectively).

**Conclusion:**

The physicians assessed presented high leadership potential in 25% of the cases, requiring improvement in the performance domains, such as interpersonal relationship, problem solving, decision-making, personal organization and time management.

## RESUMO

**Objetivo:**

Avaliar o potencial de liderança de médicos ativos de um hospital público na cidade de São Paulo.

**Métodos:**

Estudo-piloto descritivo, no qual foram selecionados aleatoriamente 40 médicos assistentes e residentes para receberem o *link* com convite eletrônico da empresa Caliper Estratégias Humanas do Brasil. Aos que o aceitaram, foi encaminhado o *link* para preenchimento de avaliação de personalidade focada no trabalho, composta por 112 alternativas relativas a 21 domínios de 4 áreas de desempenho. De acordo com Questionário Perfil Caliper, as medidas ipsativas expressas em percentual são distribuídas em uma escala do tipo Likert, e são determinadas três categorias em relação às tendências comportamentais no trabalho: necessidade de aprimoramento, potencial moderado e alto potencial.

**Resultados:**

A taxa de adesão dos médicos convidados ao estudo foi de 47,5% (19 médicos). No domínio liderança, a necessidade de aprimoramento ultrapassou 30% dos médicos avaliados. No relacionamento interpessoal, apenas 18,4% dos médicos assistentes necessitavam de aprimoramento e, no grupo dos médicos residentes, 37% necessitavam aprimoramento. Para resolução de problemas e tomada de decisões, as percentagens de necessidade de aprimoramento foram semelhantes (12,6% *versus* 14%). Na avaliação da organização pessoal e da administração do tempo, obtivemos percentagens semelhantes entre médicos assistentes e residentes, com necessidade de aprimoramento (14% nos dois grupos) e alto potencial nestas áreas (18,4% e 20% para médicos assistentes e residentes, respectivamente).

**Conclusão:**

Os médicos avaliados apresentaram alto potencial de liderança em um quarto dos casos, necessitando aprimoramento nas áreas de desempenhos, como relacionamento interpessoal, resolução de problemas, tomada de decisão, organização pessoal e administração do tempo.

## INTRODUCTION

For a more efficient growth in the health system, it is necessary to have some way to increase the number of qualified specialists in high demand areas. These specialists must have the capacity to carry out their clinical activities, and be able to fulfill roles in business leadership to support models of sustainable health system. ^(^ ^1^ ^)^

Medical teaching provides extensive training for the development of clinical skills; however, little attention is given to the development of leadership skills. The current training of physicians may, on the contrary, discourage the development of leadership skills. ^(^ ^2^ ^,^ ^3^ ^)^

The current challenges in the health sector create the growing need for good leadership. Nevertheless, leaders today are chosen only by academic or clinical criteria. ^(^ ^4^ ^)^ To improve this situation, the leadership skills of physicians have been more studied by researchers. ^(^ ^4^ ^,^ ^5^ ^)^

In the industrial sectors, training for leadership is well established, while in health organizations the same interest has not been observed over the past decades. ^(^ ^4^ ^)^ Only recently there has been a consensus on the importance of developing leader physicians.

Goodall evaluated the best 100 hospitals in the United States in the fields of cancer, digestive disorders and heart diseases, found predominance of hospital management by physicians with leadership training. ^(^ ^5^ ^)^

Greater awareness of the need for effective medical leadership has led to a significant interest in understanding how to better identify and prepare physicians with the potential to be leaders. ^(^ ^6^ ^)^

There is evidence that, in order to identify and enhance leadership potential, personality-based approaches are highly effective in various organizational contexts. ^(^ ^7^ ^-^ ^9^ ^)^ Several studies showed that personality is associated with objective measurements of performance at work, proficiency for training, and success in teamwork. ^(^ ^7^ ^,^ ^10^ ^)^ Personality assessment is an important method of predicting behavior at work.

Personality in the context of organizations has also been significantly related with productive behaviors (such as good customer service, practice of citizenship, creativity, and effectiveness in sales), as well as counterproductive ones (absenteeism, high turnover, and non-compliance with schedules. ^(^ ^11^ ^,^ ^12^ ^)^

The Caliper Profile Questionnaire (CPQ) proposed by Greenberg et al., has been used and extensively researched in recent years. ^(^ ^13^ ^-^ ^15^ ^)^ The current version of the CPQ presents 45 items that identify and measure key leadership behaviors that have been associated with individual and organizational success. It is a self-administered personality assessment focused on work, which asks respondents to indicate which personal statements are more or less similar to theirs (semi-ipsative tetrad format).

Despite its importance, the CPQ has not yet been used in Brazil to study the potential for medical leadership.

## OBJECTIVE

To evaluate the leadership potential of physicians working at a public hospital in the city of São Paulo.

## METHODS

A descriptive pilot study, in which 40 active surgeons (residents and assistants) of *Santa Casa de Misericórdia de São Paulo* , a teaching hospital, were randomly selected in 2015. All physicians received an electronic message inviting them to participate in the research, with the detailed procedures and the Informed Consent Form (ICF), which was signed and returned by those who accepted to be part of the study. Each physician who accepted to participate in the study returned the ICF and received a new electronic message with the link to fill out the CPQ online. This study was previously approved by the Internal Review Board of *Santa Casa de Misericórdia de São Paulo* , CAAE: 47573015.7.0000.5479.

All those who did not want to participate in the study or did not return their responses were excluded.

Caliper Profile Questionnaire ^(^ ^13^ ^-^ ^15^ ^)^ is authorized for use in research and publishing in scientific journals in Brazil by the company Caliper Brazil. The assessment includes Likert-type items, which evaluate openness, sensitiveness, and flexibility, as well as a multiple-choice section to test abstract reasoning skills.

In total, there are 112 statements related to 21 behavioral tendencies, distributed in four areas of performance. Each area with its behavioral tendencies is detailed below. In the leadership area, the following parameters were analyzed: assertively presents goals, strategies, perceptions and tactics; is persuasive in selling ideas, gaining support and commitment; offers clear guidance; sets standards and defines expectations; delegates responsibilities; negotiates to achieve mutually acceptable results; and provides training, counseling and feedback to develop others. In the area of interpersonal relationship, the following items were analyzed: initiates contacts; maintains relationships; cooperates with others; listens and tries to adapt based on information received and other points of view; and accepts and responds well to instructions. In the area of problem-solving and decision-making, the following points were analyzed: identifies problems, issues and opportunities; examines less obvious issues and their main causes; evaluates and considers alternative situations; and develops a plan for implementation and making decision. In the area of personal organization and time management, the parameters analyzed were: establishes goals, objectives and priorities; works well within established rules, standards and procedures; manages time and priorities efficiently; develops the activities as to guarantee the completion of the tasks within the appropriate deadlines; and works to ensure accuracy and efficiency in task accomplishment.

Bivariate correlation analysis was employed to determine the magnitude of the relation between personality dynamics, leadership style, and competency-based performance. Upon completion of the evaluation, participants received a report that included the summary of their individual results.

The results of this evaluation were expressed in percentages and distributed in the Likert scale from 0 to 100%: which classifies individual variables into three categories, ranging from (1) needs improvement; (2) moderate potential; and (3) high potential regarding behavioral tendencies.

We analyzed the profile of the physicians evaluated in relation to hierarchy and results of the percentage scores of behavioral tendencies in the areas of sample performance associated to the questionnaire administered.

## RESULTS

Of the 40 surgeons invited among experienced physicians and residents, 19 (47.5%) effectively completed the questionnaire. Of the total invited, 26 (65%) were part of the staff of the Department of Surgery of *Santa Casa de São Paulo* , and 13 (50%) participated in the project. A total of 14 residents were invited and 6 (42.8%) took part in the project.

Behavior trends in the performance areas are presented in tables 1 to 4 [Table t2] [Table t3] [Table t4].

**Table 1:** Leadership performance of assistant physicians and resisdents

Performance areas: leadership	Needs improvement		Moderate potential		High potential	
		
	Assistant physicians (%)	Residents (%)	Assistant physicians (%)	Residents (%)	Assistant physicians (%)	Residents (%)
Assertively presents goals, strategies, perceptions, and tactics	31	17	38	33	31	50
Is persuasive in selling ideas, gaining support and commitment	31	17	38	50	31	33
Offers clear guidance, establishes goals, and defines expectations	31	17	31	50	38	33
Delegates responsibilities	38	50	31	33	31	17
Negotiates to get to mutually acceptable results	31	50	54	50	15	0
Offers training, guidance, and feedback to develop others	15	50	62	50	23	0
Total average	29.5	34	42	44	28	22

**Table 2 t2:** Interpersonal relationship performance of assistant physicians and residents

Performance areas: interpersonal	Needs improvement		Moderate potential		High potential	
		
	Assistant physicians (%)	Residents (%)	Assistant physicians (%)	Residents (%)	Assistant physicians (%)	Residents (%)
Initiates contacts	46	50	39	33	15	17
Maintains relationships	15	33	85	67	0	0
Cooperates with others	8	34	61	33	31	33
Listens and tries to adapt based on information received and other stand points	15	67	62	16	23	17
Accepts guidance and responds well	8	0	31	67	61	33
Total average	18.4	37	55.6	43	26	20

**Table 3 t3:** Solving problems and decision making performance of assistant physicians and residents

Performance areas: problem solving and decision making	Needs improvement		Moderate potential		High potential	
		
	Assistant physicians (%)	Residents (%)	Assistant physicians (%)	Residents (%)	Assistant physicians (%)	Residents (%)
Recognizes problems, issues, and opportunities	8	17	46	83	46	0
Analyses less evident issues and their main causes	8	17	54	33	38	50
Evaluates and considers alternative situations	8	17	69	83	23	0
Develops implementation plan	8	0	54	83	38	17
Decision making	31	17	46	33	23	50
Total average	12.6	14	53.8	63	33.6	23

**Table 4 t4:** Personal organization and time management performance of assistant physicians and residents

Performance areas: personal organization and time management	Needs improvement		Moderate potential		High potential	
		
	Assistant physicians (%)	Residents (%)	Assistant physicians (%)	Residents (%)	Assistant physicians (%)	Residents (%)
Defines goals, objectives, and priorities	54	67	46	33	0	0
Follows established rules, policies and procedures	0	0	77	67	23	33
Manages efficiently time and priorities	8	0	69	83	23	17
Develops activities as to accomplish tasks within the adequate deadlines	8	0	69	83	23	17
Works to guarantee accuracy and efficacy in accomplishing tasks	0	0	77	67	23	33
Total average	14	14	67.6	67	18.4	20

In the group of assistant physicians, 70.5% had moderate and high potential for leadership, and 85% had moderate and high potential to offer training, advice and feedback to develop other professionals. Concerning the need for improvement, 38% presented problems in delegating responsibilities.

In the group of residents, considering the questionnaire on leadership, 66% of the evaluated physicians had moderate and high potential for leadership, and 83% had a moderate and high potential to assertively present goals, strategies, perceptions, and tactics, besides being persuasive in selling ideas, gaining support, and commitment. However, 50% needed improvement in delegating responsibilities, negotiating for mutually acceptable results, and providing training, counseling, and feedback to develop others.

In the analysis of the interpersonal relationship of assistant physicians, 81.6% presented moderate or high potential. In the subanalysis, 92% had moderate or high potential for cooperation with others, but 46% presented difficulties in initiating contacts.

In the group of residents, 63% had moderate or high potential for interpersonal relationship, and 100% had moderate or high potential in accepting and responding well to instructions. However, 50% needed improvement in initiating contacts, and 67% in listening and trying to adapt themselves based on information received.

In the item referring to problem solving and decision making in the group of assistant physicians, 87.4% presented moderate or high potential. In the general analysis, 92% of the assistant physicians had moderate or high potential to identify problems, issues and opportunities, as well as to analyze less evident issues and their main causes, and also to evaluate and consider alternative situations, and develop an implementation plan.

Among the residents, 86% presented moderate or high potential. High potential regarding identification of problems, questions and opportunities, as well as evaluation and consideration of alternative situations were not found in this group.

Regarding personal organization and time management, 86% of assistant physicians evaluated had moderate or high potential, and 54% needed improvement in establishing goals, objectives and priorities; 100% worked well according to established rules, standards and procedures. In the group of residents, 87% presented moderate or high potential for personal organization and time management. Only in the item related to establishing goals, objectives and priorities, 67% needed improvement; 100% had moderate or high potential for other items.

## DISCUSSION

Due to many economic, social and demographic factors, there is a tendency to increase the number of people seeking better health care, willing to pay more for this service, not only in the Brazilian health system, but also in other countries.

Brazil has the most expensive health market in Latin America, besides a greater tendency to annual increase (13.2%) due to the greater demand for private health care, driven by the rapid growth of the country. This growth has also led to a substantial increase in private investment in the health system, with a significantly increasing number of public-private partnerships. ^(^ ^16^ ^)^

Kisalaya et al., ^(^ ^16^ ^)^ conducted extensive analysis on the potential increase in demand for physicians acting in different medical fields. These analysts estimate that, by 2022, there will be approximately 45% increase in the need for active clinicians in Brazil, and this need should be greater in therapeutic areas or in specialties for chronic or end-stage patients (85%), internal medicine (71%), and general surgery (55%). These figures rise substantially when the need for specialists with deep knowledge in health is considered, such as research and pharmaceutical manufacturing.

Another trend that continues to have a significant impact on human capital in the Brazilian health sector is the movement toward increasing efficiency through consolidation. There is a large number of strategic merges or acquisitions involving hospitals, clinics, laboratories (imaging and diagnostic), manufacturers of pharmaceuticals and medical devices, and wholesale drug industries.

For all the above mentioned reasons, it is important that people with the right profile are well trained to lead the processes. Among the different tools to assess personality in organizational settings, 12 were exhaustively reviewed by Prewett et al., ^(^ ^11^ ^)^ regarding their psychometric characteristics, especially the CPQ, whose administration evaluates personality traits and cognitive skills, ^(^ ^17^ ^)^ and is one of the most accurate ways to assess the compatibility of people with specific job positions. In order to match the right person to the right job in a wide range of occupations, this instrument has been studied, updated and applied consistently over the past 50 years.

The items encompass positive and negative personality traits and cognitive skills, such as aggressiveness, assertiveness, helpfulness, caution, ego-drive, ego strength, empathy, energy, external structure, flexibility, gregariousness, ideas trends, level-headedness, openness, exposure to risks, internal structure, sensitivity, skepticism, sociability, stress tolerance, precision, sense of urgency and reasoning ability. These definitions are found in Resick et al. ^(^ ^17^ ^)^ It is an ipsative measurement, that is, self-referential, which, once the evaluation is done, will refer the subject to specific training.

It has been suggested that the placement of trained physicians in leadership positions may result in improved hospital performance and patient care. Some hospitals, such as University Hospital Cleveland Medical Center, in Cleveland, Ohio, and Mayo Clinic Hospital, in Rochester, introduced medical leadership training courses, and leadership education was incorporated into medical education in 2007. ^(^ ^5^ ^)^

Stoller ^(^ ^4^ ^)^ shows the factors that hinder the development of medical leaders: no tendency for collaboration and continuous training; organization of the health system presenting challenging environments; traditional leadership criteria associated with clinical and academic skills, rather than leadership skills; and, finally, little attention given to the training of physicians, regardless of their competencies. Stoller further suggests four characteristics of physicians and their training that conspire against the need to collaborate with leadership skills training: (1) training through hierarchy, often through extensive subordination; (2) extensive personal assessment for individual performance rather than group performance; (3) sense of leadership extrapolated out of the work environment; and (4) inability to think of differential medical diagnoses at the administrative level, preferring one single diagnosis.

The definition of competencies of an ideal medical leader will guide the syllabus and format of medical leadership development programs. Xirasagar et al., ^(^ ^18^ ^)^ presented four key elements to transform a leader: idealized influence, motivational inspiration, intellectual stimulation, and individualized communication.

Taylor et al. ^,(^ ^19^ ^)^ proposed five general competencies necessary for an effective leader physician: knowledge, emotional intelligence, vision, institutional altruism, and humility. ^(^ ^20^ ^)^ Kotter ^(^ ^21^ ^)^ has already demonstrated that corporate success does not wait for leaders to appear. Corporations actively seek out people with leadership potential and expose them to career experiences designed to develop their leadership potential.

The best strategy is yet unknown, as well as the training format in which leadership skills can be encouraged; however, studies that may begin to answer some of these questions are crucial for the enthusiasm. The financial resources for research in this area are still little explored. The organizations that develop answers and implement solutions to the issues will likely design the future of the health system.

This study brings partial results of a larger study and we have future objectives of conducting new studies, with a larger and more diversified sample of physicians from the entire Brazilian health system. This is the first systematic attempt to self-assess fundamental competencies, performance behaviors, and personality dynamics more closely related to medical leadership.

Only 50% of assistant physicians and 42.8% of residents invited participated in the study, even being aware that they would receive valuable information for self-knowledge related to leadership potential, motivational factors, and areas of great benefit for continued professional development. In view of the feedback offered, we considered little participation, which could be due to the fact that physicians in the studied hospital, even young ones (residents, *e.g.* , are being trained only for an eminently clinical practice, in which the patient is diagnosed and treated, as if this practice did not have to be conducted in highly institutional and organizational environment. On the contrary, organizational variables, increasingly necessary for the right person to perform the most appropriate role for their profile in health organizations, are slightly or not addressed in medical training. As a consequence, quite a few physicians did not join the study that would bring them pinpointed benefits to understand how to develop their medical career. Another fact that may have discouraged participation in the study refers to the extremely delicate economic and political moment the entire Brazilian health system - not only public, but also private – is going through. The hospital where this study was carried out is also inserted in this context. This scenario, of course, impacts on the motivation of these physicians to search information for their professional development in organizational aspects.

There is a moderate potential in the physicians evaluated, which may be better developed in some performance areas, such as interpersonal relationships, problem solving, decision making, personal organization, and time management. Regarding the potential for leadership, the need for improvement exceeds 30% of evaluated physicians, although the frequency of high potential for leadership was very close (25%).

## CONCLUSION

One fourth of the physicians evaluated in this study demonstrated a high leadership potential, needing improvement in some performance areas, such as interpersonal relations, problem solving, decision making, personal organization, and time management.
